# Use of Prescribed Psychotropics during Pregnancy: A Systematic Review of Pregnancy, Neonatal, and Childhood Outcomes

**DOI:** 10.3390/brainsci9090235

**Published:** 2019-09-14

**Authors:** Catherine E. Creeley, Lisa K. Denton

**Affiliations:** Department of Psychology, State University of New York at Fredonia, Fredonia, NY 14063 USA; denton@fredonia.edu

**Keywords:** maternal/fetal, neonatal, pregnancy, drugs, teratogenicity, behavior

## Abstract

This paper reviews the findings from preclinical animal and human clinical research investigating maternal/fetal, neonatal, and child neurodevelopmental outcomes following prenatal exposure to psychotropic drugs. Evidence for the risks associated with prenatal exposure was examined, including teratogenicity, neurodevelopmental effects, neonatal toxicity, and long-term neurobehavioral consequences (i.e., behavioral teratogenicity). We conducted a comprehensive review of the recent results and conclusions of original research and reviews, respectively, which have investigated the short- and long-term impact of drugs commonly prescribed to pregnant women for psychological disorders, including mood, anxiety, and sleep disorders. Because mental illness in the mother is not a benign event, and may itself pose significant risks to both mother and child, simply discontinuing or avoiding medication use during pregnancy may not be possible. Therefore, prenatal exposure to psychotropic drugs is a major public health concern. Decisions regarding drug choice, dose, and duration should be made carefully, by balancing severity, chronicity, and co-morbidity of the mental illness, disorder, or condition against the potential risk for adverse outcomes due to drug exposure. Globally, maternal mental health problems are considered as a major public health challenge, which requires a stronger focus on mental health services that will benefit both mother and child. More preclinical and clinical research is needed in order to make well-informed decisions, understanding the risks associated with the use of psychotropic medications during pregnancy.

## 1. Introduction

According to the World Health Organization [1], high-income countries dominate global pharmaceutical consumption—16% percent of the world’s population living in high-income countries accounts for over 78% of global expenditures on medicines; wealthy countries purchase and consume approximately 90% of total medicines (by value). The USA, in particular, carries the largest market share, which rose from 18.4% in 1976 to over 52% by 2000 [2]. According to a 2014 study conducted by the U.S. Centers for Disease Control, almost half of the U.S. population reported having taken prescription drugs in the past month. Additionally, the study found that many individuals take multiple medications; 22% reported taking three or more medications in the past 30 days, and 11% reported five or more in that same period of time. The most frequently prescribed classes were analgesics, antihyperlipidemics, and antidepressants [3]. The rise in prescription drug use most certainly includes a particularly sensitive class of patient—the pregnant woman. A recent study shows that during the last 30 years, the use of prescription drugs by pregnant women has grown by more than 60%; almost 90% of women report taking at least one medication, and 70% report taking a prescription drug [4]. 

Of particular concern are psychotropic medications, which may differentially affect fetal neurodevelopment compared to other agents. Among women who had been pregnant in the past year in the U.S. (including currently pregnant and postpartum women), 25.3% would meet criteria for a psychiatric disorder [5]. Worldwide, it is estimated that about 10% of pregnant women and 13% of women in the postpartum period experience a mental disorder. In a recent U.S. study of over 340,000 women who had a live birth between 2006 and 2011, Hanley et al. [6] found that approximately 10% of women were prescribed a psychotropic drug during pregnancy, and this average varied from 6% to 15% between states. The prescription rate was found to be stable over that six year period, with the most commonly prescribed psychotropics including selective serotonin uptake inhibitors (SSRIs), and benzodiazepine (BZDs) or benzodiazepine-like drugs, either alone or in combination, prescribed for depression and/or anxiety. Because childbearing age often coincides with the onset of mental illness, the fetal effects of psychotropic drugs used to treat mentally ill women are of particular importance. Most disorders, such as depression, anxiety disorders, bipolar disorder, and schizophrenia require some form of medical management using therapeutic drugs during pregnancy.

In most discussions involving the assessment of the risk–benefit ratio for drug use during pregnancy, risk is assessed according to observed adverse pregnancy outcome (i.e., teratogenicity, preterm birth and low birth weight), and has not typically considered long-term neurobehavioral consequences (i.e., developmental delays and learning disabilities, or “behavioral teratogenicity”) that persist into childhood and potentially beyond. The neurodevelopmental risk of psychotropic medications to the fetus deserves particular attention. When evaluating the safety of medication use during pregnancy, all of the risks associated with prenatal exposure need to be assessed, including teratogenicity, neurodevelopmental effects, toxicity, and behavioral teratogenicity. The objective of this paper is to provide a comprehensive report of the most recent results and conclusions of original research and reviews, respectively, which have investigated the short- and long-term impact of psychotropic drugs commonly prescribed to pregnant women for psychological disorders, including mood, anxiety, and sleep disorders.

### 1.1. Disorders during Pregnancy

#### 1.1.1. Depression Disorders

Mood disorders include major depressive disorder, bipolar disorder, persistent depressive disorder, cyclothymia, and minor depression. Women have higher rates of mood disorders than men in almost every age group [7]. The results of studies on mood disorder symptomology during pregnancy vary, and may even contradict each other. For example, it has been reported both that pregnant women experience highest level of depressive symptoms between 34 and 38 weeks gestation [8], and also that there is an improvement in mood during the second and third trimesters [9,10]. However, neither of these studies considered the role of medication use, which illustrates that results are difficult to interpret if it is unclear whether changes in symptomology are the result of the start of treatment, dose/drug modification, or medication discontinuation because of the pregnancy. During pregnancy, depression has a point prevalence of 15.5% in early and mid-pregnancy, 11.1% in the 3rd trimester, and 8.7% in the post-partum period [11]. However, the interpretation of the results of studies on the prevalence and course of depression during pregnancy is also complicated by varied methodologies and procedures, and differences in study populations. The research does show that pregnancy does not protect or alleviate depression symptoms, and may be, in fact, a period of high vulnerability [12].

Therefore, it appears that the risks of untreated mood disorders during pregnancy are great enough to argue for pharmacotherapeutic treatment during pregnancy (see Table 1). Therefore, research on the effects of drug treatment during pregnancy is of particular importance to the short- and long-term health of mothers and babies. The risk for relapse of depression is well-described in patients who discontinue antidepressant medication, and is a major concern because it may result in an increased risk of poor prenatal care and maternal/fetal nutrition, pregnancy complications, and postpartum depression [13,14]. Also, it has been hypothesized that the hypothalamic-pituitary-adrenal dysregulation associated with untreated depression could have adverse effects on fetal health and the developing child [15]. 

#### 1.1.2. Anxiety Disorders

Anxiety disorders are also more common among women, and include generalized anxiety disorder, panic disorder, social anxiety, and specific phobias. Obsessive compulsive disorder (OCD), while not technically an anxiety disorder, has anxiety as a main symptom, is often comorbid with anxiety disorders, and is treated with similar medications. In fact, many people diagnosed with OCD have a comorbid disorder or mental illness (i.e., depression) that complicates treatment and may lead to the use of combination drug therapy. The course of illness during pregnancy varies by disorder, and some disorders are more researched than others. For example, little research has been conducted on the course of post-traumatic stress disorder or generalized anxiety disorder during the perinatal period. With respect to panic disorder, pregnancy can be protective against symptoms, and it is speculated that hormones such as progesterone may have anxiolytic effects. In a recent Canadian study of 310 women, conducted from 2007 to 2010, the prevalence of anxiety disorder during pregnancy and the early postpartum period was 15.8% and 17.1%, respectively, and the prevalence of OCD exceeded that for adults aged 18–64 [49]. Less than 5% of women were comorbid for anxiety and depression. For some women, symptoms may worsen postpartum, in a matter of days after childbirth [50]. OCD symptoms may also appear for the first time during pregnancy [51,52], with onset particularly likely in the first and second trimesters [53]. Also, pregnancy may correspond to a worsening of symptoms in those with pre-existing cases. In one survey of women with OCD, 33% reported that pregnancy exacerbated their symptoms [52]. For example, OCD may manifest as intrusive and distressing obsessions about harming the fetus/infant [54].

#### 1.1.3. Bipolar Disorder

There is very little, if any, information on how or whether Bipolar Disorder (BPD) changes over the course of a pregnancy. There is evidence that pregnancy could be protective against BPD symptoms [55,56]. However, subsequent retrospective research suggests that pregnancy is a period of substantial risk for recurrence, with estimates as high as 50% [34,57,58]. High rates of relapse have been observed in women with BPD. In a study [59] examining recurrence of symptoms during pregnancy, 70.8% of the women were reported to experience at least one mood episode, and the risk of recurrence was more than two times higher in women who discontinued treatment compared to those who maintained treatment. A subsequent study of women in the U.S. and Italy found that among women with mood disorders, the risk for illness was the highest during the postpartum period, especially for women with BPD [56]. A major conclusion of these studies was that discontinuation of medication use may be the most important predictor for recurrence of symptoms in pregnant women. Due to the severity of consequences of forgoing treatment and/or relapse, prenatal exposure to drugs used to treat BPD is likely to occur either throughout or during the first and third trimester.

#### 1.1.4. Sleep Disorders

Short-term, chronic, and other types of insomnia are the three major categories based on diagnostic criteria that include difficulty falling asleep, difficulty staying asleep, or early awakening. For a sleep disorder diagnosis, symptoms must be associated with impaired daytime functioning and occur at least three times per week for at least one month. Insomnia, and the use of prescription sleep aids, is more common in women compared to men [60]. Sleep disorders include insomnia, restless leg syndrome, narcolepsy, and sleep apnea. Disrupted sleep affects the daily life of millions of Americans—over 35% of Americans in every age group report unintentionally falling asleep during their day, and 2%–7% report falling asleep at the wheel. Women are more likely to report problems with sleep compared to men [61,62], and sleep disturbances are common in pregnancy, with over 80% of women reporting disturbed sleep and 15% of women developing Restless Legs Syndrome (RLS) during pregnancy [63,64]. A recent meta-analysis of sleep quality during pregnancy verified that problems with sleep are common during pregnancy, and also found that sleep quality may decline in the third trimester. Mood and gestational age were also identified as important covariates in the extent of sleep disturbances experienced by women throughout pregnancy. The authors concluded that sleep disturbances during pregnancy could be characterized as mild, and should be expected, but that complaints about severe sleep problems should be taken seriously and that intervention may be required, as serious sleep problems are associated with an increased likelihood of preterm birth, longer labor, cesarean delivery, and prenatal and postpartum depression [65].

Please refer to Table 2 for the overall prevalence, age of onset, and pregnancy prevalence rates for each of the disorders described above.

### 1.2. Pharmacotherapy

#### 1.2.1. Depression

Antidepressant (AD) use is increasing at an alarming rate in the U.S. In a recent national health study, antidepressants were reported to be the most frequently used prescription drug by 18–44 year-olds [75]. The Center for Disease Control (CDC) reported that, each year, from 2008 to 2013, 15.4% of reproductive-aged women filled a prescription for an antidepressant from an outpatient pharmacy. Roughly three-quarters of these women filled prescriptions for only one type of antidepressant. The most common prescriptions were for sertraline (3.3%), bupropion (2.7%), citalopram (2.6%), escitalopram (2.5%), and fluoxetine (2.3%). From 1988 to 1994, and again through the period 2005–2008, the rate of antidepressant use in the United States increased, overall, by 400%. Women are 2½ times as likely to take antidepressant medication as males, and more likely to take medication at every level of depression. Data analysis from 1996 to 2005 shows that antidepressant use during pregnancy has steadily increased, with the largest increase seen in the use of SSRIs. Nearly 8% of pregnant women were prescribed antidepressants during the years 2004 and 2005. Within this sample, the most common AD was the SRI (6.7%), followed by other ADS (1.3%) such as Bupropion (0.7%), Venlafaxine (0.3%) and Trazodone (0.3%) [76,77]. Currently, the first-line treatment for depressive disorders is one of the serotonin reuptake inhibitors (SRIs), which have fewer side effects and are considered to be safer and more effective than the earliest ADs, tri- or tetra-cyclics, or monoamine oxidase inhibitors (MAOIs). The five SRIs approved for use for depression, in the order of United States Food and Drug Administration (FDA) approval, are fluoxetine (Prozac), sertraline (Zoloft), paroxetine (Paxil), citalopram (Celexa), and escitalopram (Lexapro). Sertraline is becoming a popular treatment for maternal depression, and is estimated to affect up to approximately 6.2% of pregnancies [77,78]. Venlafaxine is a serotonin and norepinephrine reuptake inhibitor (SNRI) first approved for treatment of depression, followed later by approval for generalized anxiety disorder. Atypical or “newer generation” ADs are prescribed to treat depression; the FDA approved atypicals include: burproprion (Wellbutrin), mirtazapine (Remeron), nefazodone (serzone), trazodone (desyrel), and vortioxetine (Trintellix). Because the age of onset and diagnosis for women is typically during reproductive years, drug treatment before, during and after pregnancy is likely. The FDA safety data and warnings for ADs are summarized in Table 3 [79]. 

#### 1.2.2. Anxiety and Obsessive-Compulsive Disorder (OCD)

The most common classes of medications use to treat anxiety disorders are ADs, benzodiazepines (BZDs) and beta-blockers. The SRIs are the first-line choice for treatment of anxiety disorders, but BZDs are still prescribed [80]. The disorders outside of depression that SRI/SNRIs were approved to treat, listed in order of approval, are as follows:

- 1987—fluoxetine (Prozac): OCD, panic disorder
- 1991—sertraline (Zoloft): OCD, panic disorder, social anxiety disorder
- 1992—paroxetine (Paxil): OCD and panic disorder, social anxiety disorder, generalized anxiety disorder
- 1993—venlafaxine (Effexor): generalized anxiety disorder, panic disorder, social phobia
- 1998—citalopram (Celexa): None
- 2002—escitalopram (Lexapro): Generalized anxiety disorder

The SRIs are structurally diverse, which results in distinct pharmacological profiles, resulting in differences in potency and effect. The FDA pregnancy warnings for SRIs are listed in Table 3.

#### 1.2.3. Bipolar Disorder 

In most, if not all cases, long-term pharmacotherapeutic treatment with mood-stabilizers is indicated for bipolar disorder [81]. The classes of drugs most often used include mood stabilizers (lithium), anticonvulsants (valproate, carbamazepine, lamotrigine) and atypical antipsychotics (clozapine, risperidone, olanzapine, quetiapine, ziprasidone, aripiprazole, asenapine, and lurasidone). Currently, valproate, carbamazepine, and lamotrigine are approved for the treatment of bipolar disorder. Antipsychotic drugs are also increasingly prescribed to BPD patients across the world [82,83,84,85]. The antipsychotics most commonly used to treat BPD are rispiridone and olanzapine. A recent study of maternal use of atypical/typical antipsychotics, antieplieptics (AEDs), and lithium reported that the use of atypical antipsychotics and AEDs during pregnancy has increased, while the use of typical antipsychotics and lithium has decreased. Data showed that anticonvulsants were the most frequently used class of medication. Polypharmacy was common, mostly including combinations of anticonvulsants and atypical antipsychotics (45.5%). Other drug combinations reported included lithium and a typical antipsychotic (10.7%), typical antipsychotics and atypical antipsychotics (10.4%), typical antipsychotics and anticonvulsants (9.4%), atypical antipsychotics and lithium (7%), and lithium and anticonvulsants (4.8%). Over 12% of pregnant women in the study filled prescriptions for medications from three or more categories [86]. The FDA pregnancy risk categories and warnings for mood stabilizers, anticonvulsants, and atypical antipsychotics are summarized in Table 4.

#### 1.2.4. Sleep Disorders

Sleep and mood disorders are often comorbid, and women with mood disorders experience more sleep and circadian rhythm disturbances during both pregnancy and the postpartum period [87,88]. Changes in sleep patterns have been connected to postpartum depression, especially in women with a mood disorder. This increased risk for mood disruption due to lack of sleep in the 3rd trimester and postnatally may lead clinicians to treat sleep problems pharmacologically, thereby exposing neonates to drugs used to treat sleep problems. Until recently, barbiturates and benzodiazepines have used to treat sleep disorders. These drugs have been known to be particularly dangerous when mixed with other medications or alcohol, have abuse/addiction potential, and are not generally recommended for sleep aid use during pregnancy. The newer hypnotic benzodiazepine receptor agonists (HBRAs), such as zolpidem (Ambien), eszopiclone (Lunesta), and zalepon (Sonata), are more selective in their receptor binding compared to benzodiazepines, which non-selectively bind to multiple GABAα receptor subtypes. Zolpidem and zaleplon, for example, selectively bind to the α–1 subunit, and eszopiclone is more selective to the α-2 and α-3 subunits. Binding selectivity improves the safety profile, but HRBAs still share similar side effects and therefore may share some of the risks of use during pregnancy. Zolpidem is one of the most commonly prescribed sleep medications in the U.S., and is considered the safest sleep aid, but not necessarily for women. Recent research has found that there was a 274% increase in the number of women who went to the emergency room due to a reaction involving zolpidem, (compared to a 144% increase among men). In 2010, women accounted for 68% emergency room visits for an adverse zolpidem reaction [89]. Prevalence data on the use of HRBAs during pregnancy are scarce. However, there have been several studies conducted to investigate whether HRBAs have teratogenic effects, and researchers were able to identify thousands of women who have used these drugs during pregnancy [90,91,92]. One study conducted in Denmark on the prevalence of HBRA use during pregnancy (1997 to 2010) revealed that the use of HBRAs among pregnant women increased from 0.2% to 0.4% per year until 2010, when use decreased back to 0.2%. In 2010, zopiclone (59%), zolpidem (44%) were the only HRBAs used during pregnancy [93]. 

## 2. Materials and Methods

A literature search strategy using the Medline and PsychInfo databases, and reference lists of reviews was conducted for studies published on or before 1 August, 2019. Search terms for psychological disorders included: depression, mood disorders, anxiety, obsessive-compulsive disorder, and insomnia. Disorders were cross-referenced with terms for outcome variables, which included: pregnancy, neonatal, teratogenicity, behavioral teratogenicity, neurotoxicity, and neurodevelopment. Preclinical research using animal models and human clinical studies were reviewed. Review articles and meta-analyses were considered to be secondary sources, and were used both as references and to ensure a comprehensive search was conducted. Original, preclinical research articles were examined for evidence of short- and long-term teratogenic, neurotoxic, and neurobehavioral effects in relevant animal models. Clinical studies were reviewed and evaluated for short- and long-term outcomes that included investigations of teratogenic birth defects, adverse pregnancy outcomes, neonatal syndromes, and evidence for long-term neurodevelopmental effects that persist into childhood. Only studies published in peer-reviewed journals were included, as publication was assumed to fulfill the requirements that they be reliable and valid. All studies were examined for research quality considerations based on evaluation of the design, methods, procedures, and conclusions as based on results. Additionally, studies were included in this review irrespective of positive results; findings that were positive, negative, or null are discussed. Where possible, the results of the most recent studies are provided as a review of the latest research on each of the most common psychological disorders experienced by women during their childbearing years. 

## 3. Effects of Maternal Pharmacotherapy on Fetal, Neonatal, and Child Development

### 3.1. Tri- and Tetracyclics (TCAs) and Monoamine Oxidase Inhibitors (MAOIs)

#### 3.1.1. Fetal and Neonatal Effects: TCAs 

The questions about potential teratogenic effects of TCAs were first raised decades ago, during the thalidomide tragedy and the advent of the Lithium Registry of Babies [94], when it was first observed that imipramine caused teratogenic effects in rabbits [95]. This prompted a series of articles on the safety of imipramine during the early 1970s that were based on case reports from physicians, very small uncontrolled studies, or opinions on previous reports. While some women who used imipramine or amitriptyline therapy did experience adverse pregnancy outcomes, the general conclusion was that there was not enough evidence to declare TCAs as teratogens, and the seriousness of the mental illness of the women posed a greater risk to the health of mother and baby than the medication [96,97,98]. This new attention to the teratogenic effects of drug exposure did not appear to cause researchers to conduct well-controlled laboratory studies specifically designed to investigate the teratogenic effects of TCAs using animal models. Therefore, animal studies designed to investigate the teratogenic effects specific to TCAs are difficult to find. There are some studies that used animal models that report decreased body weight due to clomipramine exposure, but these models often used neonatal and not fetal exposure [99,100]. There are studies that have used TCAs as a study drug that reported that exposure did not cause anatomical, skeletal, or cardiac abnormalities in animal models [101]. Among the different TCAs, the results of animal studies cited by FDA labeling do show that there are significant differences among these medications in terms of their effects on fetal development (see Table 3). 

More recent studies focus on the effects of fetal exposure to ADs on neurodevelopment, because the developing nervous system is particularly vulnerable to the neurobiological influence of continuous exposure to psychoactive drugs. In vitro evidence in adult rat neurons investigating the neurotoxicity of TCA revealed a rank order of neurotoxic potential, in order from lowest to highest: desipramine < amitriptyline < trimipramine < imipramine [102]. Maciag et al. [103] conducted a study that included clomipramine and the SRI citalopram that investigated neuroanatomical changes and behavioral outcomes. The results revealed that exposure to AD drugs can disrupt the development of serotonin systems in the fetal/neonatal brain growth spurt period, which has long-term behavioral consequences. Subsequent animal models were conducted to connect the neuroanatomical impact of drug exposure during brain development on long-term behavior. 

The results of the very first, small, uncontrolled studies in humans in the 1970s suggested that there were no teratogenic effects associated with TCA use during pregnancy [96,97,98,104,105]. Subsequent studies found no greater risk of TCA-induced teratogenicity or adverse pregnancy outcomes, but did observe short-term neonatal withdrawal symptoms in infants at birth [106]. Research comparing TCAs to the newest SRI at the time (fluoxetine) did not find significant differences in the risk for teratogenic malformations, but did find a similar increased rate of miscarriage for both types of drug [107].

Currently, as the oldest of the AD drugs, TCAs and MAOIs have been on the market long enough to have developed a post-market reputation for causing more adverse side effects than newer SRIs [108]. Decades of research findings in animals and humans have led to conflicting evidence on the teratogenic risks of the TCAs and MAOIs. A consistent finding is that TCAs can increase the risk of pre-eclampsia and induce neonatal adaptation syndrome if used in late pregnancy, but these effects cannot be separated from those that accompany untreated maternal depression [109]. In a more recent comprehensive review of the effects of TCA use during pregnancy, Gentile [110] concluded that clomipramine use carries the greatest increased risk, and nortriptyline has the lowest risk, in terms of adverse events in humans. In general, MAOI drugs have long been considered as contraindicated for use during pregnancy because of the increased risk for hypertension and their interaction profile with hormones and diet significantly complicates treatment. Therefore, the focus of this part of the review will be on the TCAs. The TCAs and MAOIs approved for use in the U.S and current drug label warnings describing pregnancy risks are outlined in Table 2. 

#### 3.1.2. Long-Term Developmental Outcomes: TCAs

Prenatal TCA exposure has been linked to behavioral teratogenicity in animal models, which has been measured by assessing locomotor activity/exploration, social interaction and cognition. These measures test exploration of and habituation to novel situations, and may uncover anxiety-like behavior. Studies have shown that activity and exploration is altered in rodents prenatally exposed to TCAs and MAOIs [111,112,113,114,115,116,117]. Social interaction behaviors were also altered in male and female rats prenatally exposed to TCAs. However, the results are difficult to interpret, because researchers have reported both an increase [114,115] and decrease in social behaviors in rodents prenatally exposed to TCAs [113,117]. Studies designed to examine the effects of fetal AD exposure on serotonin circuitry that include TCAs have demonstrated that disruption of the serotonin system during neurodevelopment is associated with a pattern of maladaptive behaviors, or a “neonatal antidepressant exposure syndrome” that persists into adulthood and causes alterations in locomotor and sexual activity, drug sensitivity, and sleep. The results of this research suggest that there is a neurobiological impact of long-term antidepressant administration on the neonatal brain that may be permanent and result in permanent behavioral consequences [103,118]. 

In the 1990s, researchers set out to determine the long-term effects of prenatal exposure to ADs in studies on child development. Most studies focused on drugs of abuse, but some, such as the Motherisk initiative in Toronto, did include ADs in their program of research, and also investigated both the short-term fetal/neonatal outcomes [107] and the long-term effects of TCAs and fluoxetine on neurodevelopment of children exposed in utero. The results of this research were that there was no difference in global IQ or language development between groups of children exposed to TCAs or fluoxetine compared to unexposed controls. There were also no behavioral differences found, which included measures of mood, arousal, activity, and distractibility [119]. A systematic review of the short- and long-term effects of AD use during pregnancy concluded that exposure to TCAs and newer SRIs, collectively, was not associated with an increased risk for intrauterine death, major malformation, or long-term neurodevelopmental deficits. They did find, though, that low birth weight and neonatal withdrawal were associated with late-term exposure [120]. Subsequent, well-controlled research that focused on exposure throughout pregnancy assessed the behavior and cognition of groups of children of depressed mothers who used TCAs or fluoxetine compared to healthy controls. The results showed that TCA or fluoxetine exposure was not associated with adverse effects on cognition, language development, or temperament of young children. They did, however, find that maternal depression was associated with impaired cognitive development and language skills in their children. The authors concluded that adequate antidepressant therapy should be maintained during and after pregnancy [121]. Much of the research that followed focused on the newer ADs, known as the selective serotonin reuptake inhibitors, now collectively referred to as the serotonin reuptake inhibitors, or SRIs. 

### 3.2. Serotonin Reuptake Inhibitors (SRIs)

#### 3.2.1. Fetal and Neonatal Effects: SRIs

Animal models used to investigate the fetal/neonatal outcomes studied and observed in human TCA research began with intense focus on fluoxetine, the first SRI. Studies in rats showed that fluoxetine caused pulmonary hypertension and structural abnormalities in the vascular system [122], and an increase in postnatal mortality at the highest doses [122,123]. In a similar mouse study, a pup mortality of 81% was linked to an enlarged heart, and this was at a much lower dose [124]. However, this finding was not demonstrated in other studies using higher doses [125,126,127]. In addition to potential teratogenic effects, fluoxetine was also found to cause minor neonatal abnormalities and developmental delays [126]. 

There are few studies, compared to the fluoxetine research, that specifically evaluate the fetal and neonatal effects of the other SRIs (i.e., citalopram, escitalopram, and sertraline). In a toxicological study of sertraline that included evaluating teratogenic effects, Davies and Kluwe [128] determined that there were no teratogenic effects at maternally toxic doses, but there was, as was observed with fluoxetine, increased neonatal mortality and also decreased body weight and growth. Prompted by the findings in human studies, and the connection between serotonin signaling and development of the heart muscle, Haskell et al. [129] investigated the effects of neonatal exposure to sertraline on long-term changes in cardiac structure and function. The results showed that neonatal sertraline exposure impaired growth of the heart and caused what was termed a “small left heart syndrome” in adult mice. Venlafaxine exposure, which is a serotonin-norepinephrine reuptake inhibitor, produced results that were both similar and different from fluoxetine. Unlike fluoxetine, venlafaxine treatment did not shorten gestation, but like fluoxetine, it did reduce birth weight of pups. Neither drug had any effect on litter size or pup mortality [126]. 

Animal research that focused on the typical human pregnancy outcomes such as gestational length, birth weight, and litter size showed mixed results. Fluoxetine exposure shortened gestation time [126], decreased litter sizes [130] and lowered birth weights [123,126,127,131,132]. However, there are also multiple studies, some by the same researchers, that report no significant effects, and these differences appear to depend on differing doses, route of administration, and time of exposure during gestation [123,126,127,131]. In a 2014 review, Bourke, Stowe, and Owens [118] carefully examined the preclinical evidence on the effects of TCAs (mainly imipramine) and SSRIs (mainly fluoxetine) in various animal models. The early studies of growth, anatomical, and physiological effects provide some evidence for teratogenicity in terms of congenital malformations caused by drug exposure, but only at high doses considered to be outside of the therapeutic range in humans. Animal research that focused on pregnancy and neonatal outcomes in humans, such as in utero growth, birth weight, and fetal/neonatal morbidity and mortality, did find some adverse effects consistent with human findings, but the results were inconsistent [133].

Animal studies were followed by years of SRI research in humans that focused on evaluating fetal and neonatal effects. The results of this research have uncovered important differential risks within the SRI class of drugs (briefly outlined in Table 3). In a very recent risk–benefit assessment of maternal AD use based on the results of clinical research, Weisskopf et al. [134] concluded that SRIs should be considered as first-line agents during pregnancy—particularly citalopram, escitalopram and sertraline. Paroxetine use is discouraged because of an increased risk of congenital heart defects above that found with other SRIs. However, the SRIs considered to be the safest have been associated with an increased risk of birth defects; namely, sertraline with septal heart defects [135,136], citalopram with cardiac malformations [137], and escitalopram with musculoskeletal malformations [138]. Most recently, Bérard et al. [139] showed that first trimester exposure to sertraline was associated with an increased risk of atrial/ventricular defects and craniosynostosis, whereas non-sertraline SRIs were associated with craniosynostosis and musculoskeletal defects.

Studies on the short-term physiological and behavioral effects studied in newborns prenatally exposed to ADs converge on a consistent finding that SRIs are associated with an increased risk of preterm birth [140]. In a prospective population-based study, mothers who used SRIs were compared to those with untreated clinical depression or no/low depression symptoms. Fetal/neonatal outcomes included gestational body and head size, preterm birth, and birth size and weight. Maternal depression was associated with reduced fetal body and head growth. Prenatal SRI exposure was also associated with reduced fetal head growth, and increased the risk of preterm birth [141]. A case-control study that compared birth outcomes of pregnant women who used SRIs during pregnancy to healthy women also found that prenatal exposure to SRIs, especially at high-doses, was associated with an increased risk of preterm birth [142]. In a similar study that examined the effects of prenatal SRI exposure on gestational age and birth and birth size (weight, length, head circumference), a within-family design was used to account for genetic and environmental confounds. The results first suggested a relationship between SRI use and growth and gestational outcomes, however, the within-family analysis removed all effects of SRI exposure except for gestational age, which was lower in the exposed infants [143]. It appears that a number of studies have found a connection between antidepressant use and premature birth, independent of type of drug or confounding genetic and environmental factors.

Because a known side effect of current SRI use is sleep disruption [144,145], sleep patterns have also been studied in fetuses and neonates exposed to antidepressants. A prenatal study of different drug types in a range of doses found that SSRIs increased fetal motor activity in utero and a disruption in non-rapid eye movement (non-REM) sleep and impaired inhibitory motor control [43]. Examination of prenatally exposed neonates (1–2 days old) also showed disrupted REM sleep, autonomic control, and increased motor activity [146]. These results that show disruption in sleep and motor control are consistent with those observed in preclinical animal studies [147], but the clinical significance is not known.

One of the most consistent neonatal effects of AD drugs that is consistent is the observation that late-term exposure to TCAs and SRIs causes drug withdrawal in the newborn. The results of a series of studies by Oberlander et al. [148,149,150,151,152] showed that infants exposed to SSRIs showed altered behavioral (facial expression) and physiological (heart rate) reactivity to pain. Studies using infant development scales have shown that prenatal SSRI-exposed neonates showed deficits in habituation, social interaction, motor, and autonomic behaviors, and/or a set of clinical signs, termed “neonatal abstinence syndrome” (NAS), which includes: irritability, trouble feeding, mild respiratory distress, tachypnea, and myoclonus [153,154]. Clinical research on the long-term effects of SRI exposure on infant and child development soon followed.

#### 3.2.2. Long-Term Developmental Outcomes: SRIs

In most of the animal studies cited above that measured fetal/neonatal effects of fluoxetine exposure, long-term behavioral tests were also conducted. Vorhees [123] evaluated neurobehavioral outcomes at three developmental stages (preweaning, juvenile, adult), and found no significant effects on any behavioral tests at any stage of development. Noorlander et al. [124], however, found that neonatal fluoxetine caused permanent cardiac anomalies that were accompanied by long-term behavioral deficits demonstrated by depression- and anxiety-like behaviors that were dose-dependent

Careful studies have been conducted on what may be termed a serotonin syndrome in neonatal animals prenatally exposed to SRIs. Kiryanova et al. [155] published an extensive review of all of the animal research that has investigated the long-term neuroanatomical and behavioral effects of early fluoxetine exposure, including locomotor/exploratory activity, sensorimotor function, social interaction, learning, and memory. Studies that used outcome measures of depression- and anxiety-like behaviors were also reviewed. The overall conclusion was that it is clear that early exposure to fluoxetine can induce long-term alterations in the serotonergic and other brain systems, which may cause behavioral teratogenicity. However, taken together, the results of behavioral studies show a pattern of findings, where there are drug effects found in some experiments, but not others, even when the same behavioral test is used. The most consistent findings are that exploratory/locomotor, social, sexual, and anxiety- and depression-like behaviors are all impacted, but the implications of these effects, and the underlying neural mechanism for these behavioral changes are still not known. The authors of comprehensive reviews generally conclude that the results of animal research are difficult to interpret because of major methodological differences in the experimental designs. When the design and methods of these studies are closely examined, dose, route, and timing of administration are revealed to be important factors in producing differential results [155]. Nonetheless, it is often the case that some preclinical results in animals converge with clinical findings in human infants and children.

In a study of infants previously determined to be normal by pediatric neurologists, who were assessed by using an infant development scale at 12–40 months of age, length of exposure was linked to an increased risk for deficits in psychomotor and behavioral development. Importantly, prenatal exposure to several different SSRIs (sertraline, fluoxetine, paroxetine, or fluvoxamine) was associated with impaired psychomotor development at ages 6–40 months in a study that controlled for maternal mood, and compared antidepressant-exposed children to children born to untreated mothers with major depressive disorder [156].

Few studies have examined the long-term effects of prenatal exposure to antidepressants. An early study evaluating TCAs or fluoxetine vs. controls found no differences in global IQ or language scores, and no significant differences in mood, temperament, activity level, attention, or other behavior problems in children assessed between 16 months and 7 years of age [119]. In a connected study using a similar age range, adverse developmental and behavioral effects were found to be associated with depression in the mother, and not to prenatal exposure to ADs. GlobaI IQ was significantly and negatively associated with duration of maternal depression, and language ability was negatively associated with number of depression episodes experienced by the mother [121]. In a follow-up to the Oberlander studies on neonatal effects, Misri et al. [106] re-examined children (previously studied at 3 and 8 months old) for signs of depression, anxiety, and withdrawal behaviors when they reached 4 and 5 years of age. Children with prenatal SRI exposure were compared to children of healthy, non-medicated mothers. The results showed no behavioral differences between children with prenatal psychotropic medication exposure compared to controls. However, symptoms of maternal anxiety and depression were associated with increased reports of depression, anxiety and withdrawal behaviors in the children. In a subsequent study, Oberlander et al. [150] evaluated infants exposed prenatally to sertraline, paroxetine, or fluoxetine and found that problems with attention, aggression, and hyperactivity did not differ between groups when maternal mood was a factor, but maternal depression and anxiety was associated with increased reports of these behaviors. The most recent study of the risks of SRI exposure during pregnancy [157] found that the cumulative incidence of depression among offspring of depressed mothers exposed prenatally to SRIs was 8.2% by age 14.9 years, compared with 1.9% in the no medication group, and to 2.8% for those where medication was discontinued. Rates of anxiety, autism spectrum disorder, and attention-deficit diagnoses were comparable to rates in offspring of mothers with a psychiatric disorder who did not use medications during pregnancy. Therefore, when comparing SRI-exposed to unexposed pregnancies, the risks of adverse neurodevelopmental outcome may be elevated for children exposed to SRIs in utero.

In recent years, increases in childhood neurodevelopmental disorder diagnoses, particularly autism spectrum disorders (ASD), has prompted further investigation in relation to prenatal AD exposure. A recent population-based case control study reported a connection between ASD and prenatal AD exposure. Specifically, maternal SRI exposure during the year before delivery, and especially during the first trimester, was associated with twice the risk of an ASD diagnosis of the exposed child. Importantly, no increased risk was found for untreated mothers [158]. In another population-based study designed to evaluate the effects of ADs according to trimester of exposure that controlled for maternal depression status, researchers found that SRI use during the second and/or third trimester was significantly associated with an increased risk of ASD in the child [159]. A population-based nested case-control study in Sweden [160] investigated the role of depression and AD medication on the risk of ASD and intellectual disability. The results showed that maternal depression and medication use (either SRIs or MAOIs) was associated with an increased risk of ASD in the child, and there was no evidence of an increased risk of autism with intellectual disability. The most recent review of the evidence linking prenatal exposure to SRIs and the risk of ASDs was conducted by systematically evaluating all of the available literature and studies on the subject since 1988 [161]. It was reported that six out of eight studies have found an association between antenatal SRI exposure and an increased risk of ASD, but that there are limitations to the research that should cause us to be careful in our interpretation of this risk. The results of another meta-analysis and review concluded that there is an increased risk of ASD in children of mothers exposed to SRIs during pregnancy, but causality has not been determined, and more research is required [162]. It is also important to note that even if causality could be assumed, AD medication use during pregnancy explains less than 1% of the cases of ASD.

### 3.3. Atypical ADs

#### 3.3.1. Fetal and Neonatal Effects: Atypicals

There are very few studies that have investigated the atypical ADs for teratogenic effects. In a study designed to compare the short- and long-term effects of bupropion, fluvoxamine, citalopram, and trazodone on cocaine sensitivity during adulthood, fetal mice were exposed to an atypical AD throughout the third trimester of gestation. Only trazodone treatment affected birth outcomes (litter size and pup mortality). Compared to saline-treated pups, trazodone treatment caused a high mortality rate in pups post-weaning [163]. A subsequent related study, designed to test cocaine sensitivity that tested dose–response to bupropion alone showed that, at various doses administered during the third trimester, bupropion caused increases in locomotor activities and anxiety-like behaviors, and altered sensitivity to the effects of cocaine during adulthood. It was suggested that prenatal bupropion exposure could increase the risk of agitation, stress susceptibility and drug sensitivity in adulthood [164]. A recent study of the postnatal effects of bupropion treatment throughout gestation found that pups weighed significantly less at birth compared to controls [165].

In a meta-analysis of prospective and comparative studies conducted from 1996 to 2005 on the rates of major malformations associated with newer ADs (SRIs, and atypical ADs), only seven studies met inclusion criteria. Only studies that compared SRIs or atypicals and evaluated malformations in exposed (first trimester) vs. non-exposed groups were included in the analysis. The results showed no association between exposure to any of the newer ADs as a group and increased risk for major malformations. There was also no association between individual ADs (bupropion, fluoxetine, nefazodone/trazodone, and venlafaxine) and an increased risk of major malformations [166]. A 2009 Motherisk Program study of a large pregnancy cohort of women who used SRIs or atypical ADS during the first trimester found similar results—as a group, ADs were not associated with an increased risk for major teratogenic effects, and no single AD carried an increased risk [167]. Two more recent meta-analyses investigated the association between various ADs and pregnancy/neonatal outcomes. The authors of the first study [168] conducted a systematic review and meta-analysis of studies designed to evaluate the association between AD use (any AD drug) and adverse pregnancy (spontaneous abortion, prematurity) and birth outcomes (gestational age, birth weight, and APGAR scores). While the effect size was small, there was a statistically significant association between fetal AD exposure and decreases in gestational age, birth weight, and APGAR scores. There was also an increased risk of premature birth in AD-exposed pregnancies. In a subsequent study that added eight more studies published after 2010, Huang et al. [169] further evaluated the pregnancy and birth outcomes in women exposed to any AD during pregnancy. The results of this study suggested that studies with negative findings on preterm birth may have not been published, but that AD exposure was associated with significantly increased risks for low birth weight and preterm birth. Evaluation of previous research also suggests that dose, timing and duration of AD exposure mediates risk, where, not surprisingly, full term exposure and use of higher doses carries the greatest risk.

#### 3.3.2. Long-Term Developmental Outcomes: Atypicals

In the study described above [163] that compared bupropion, fluvoxamine, citalopram, and trazodone, researchers tested locomotor activity, anxiety-like behavior, and sensitivity to the reinforcing effects of cocaine in adult mice exposed during the end of gestation. Only bupropion-treated mice showed changes in locomotor behaviors compared to controls, and an increased sensitivity to the reinforcing effects of cocaine. These results suggest that bupropion exposure late in pregnancy may increase the susceptibility to stress and cocaine reward that persists into adulthood. In a study that study investigated postnatal AD exposure (SRIs and bupropion), behavioral tests were conducted to assess sensory response, social, and sexual behaviors. The results showed that AD exposure was associated with decreased exploratory and social behaviors. SRIs, but not bupropion, disrupted male sexual behavior. The authors concluded that early exposure to ADs causes sensory and social abnormalities that parallel those seen in the human condition of autism spectrum disorder [170].

The long-term effects of fetal exposure to mirtazapine were investigated using behavioral tests to assess long-term changes in locomotor activity, emotional reactivity, and learning ability. The results showed that AD-exposed mice were significantly different compared to controls on measures of locomotor activity, and at the highest dose, exposed animals showed anxiety-like behaviors and learning deficits [171].

There are no human studies that have investigated the long-term neurobehavioral effects of fetal exposure to atypical ADs specifically.

### 3.4. Conclusions: AD Use during Pregnancy

A review of the preclinical and clinical research reveals that there is convergent data on factors that connect the experimental animal and non-experimental human data, which includes the acute effects of placental transfer, presence in breast milk, low birth weight, neonatal syndrome (jittery, abnormal motor activity), and sleep disturbances. Research on the short-term effects of SRIs in both animals and humans has largely been replaced by studies investigating long-term neuroanatomical and behavioral outcomes, but long-term effects are difficult to assess due to fewer animal studies and lack of well-controlled research in humans. 

There have been several comprehensive reviews of the human studies that have assessed the effects of ADs on both short- and long-term developmental outcomes [172,173,174]. Most of the studies are prospective/retrospective or case control studies, with no truly experimental, randomized controlled trials. Similar to problems within the animal research, there are major limitations inherent to human studies that include small sample sizes, lack of adequate control of confounding factors, and lack of appropriate treatment groups. Despite these challenges, more studies than not have found adverse short-term fetal or neonatal effects associated with AD use during pregnancy, including congenital malformations, miscarriage, preterm birth, low birthweight, and poor neonatal adaptation. Maternal use of TCAs or SRIs throughout gestation does not appear to cause serious deficits in cognition, language development, or the temperament of young children. Importantly, studies that include untreated maternal depression as a variable have found that the mothers’ depression alone is a significant factor, and is linked to impaired cognitive and language development in their children. The association between fetal exposure to antidepressants during gestation and later development of ASD is still controversial. Studies on the etiology of depression have implicated genetic predisposition, environmental risk factors, and maternal depression as the most important factors in short- and long-term outcome for children born to mothers with a depression disorder. However, there is also reason to suspect that psychoactive drug exposure, especially long-term and at higher doses, could cause permanent structural changes in the brain to serotonergic systems that has long-term consequences and could contribute to developmental delays and behavioral disorders in childhood. 

The overall conclusion appears to be that prenatal exposure to either maternal depression or AD drugs carries at least short-term risks to the developing fetus, but there is not enough research on the long-term cognitive effects and behavioral development, so existing information is simply too limited to accurately and fully determine risk.

### 3.5. Benzodiazepines (BZDs) and Hypnotic Benzodiazepine Receptor Agonists (HBRAs)

#### 3.5.1. Fetal and Neonatal Effects: BZDs and HRBAs

There are decades of research on BZDs using rodent models to investigate the short- and long-term neurobehavioral effects of early exposure to BZD drugs that include diazepam, clonazepam, and lorazepam. An early review [175] of BZD research on rat development concluded that adverse effects on physical development were found only at extremely high doses, and also that studies that found evidence for any long-term behavioral effects after early exposure contained methodological problems that could have confounded the results. 

A recent study investigating the teratogenic effects of benzodiazepine exposure found no evidence for an increase in congenital malformations in children exposed to BZDs and HRBAs during the first trimester of pregnancy [92]. A 2013 review of studies investigating the teratogenicity of BZDs found that, for data published during the last 10 years, there was no evidence against prescribing BZDs during the first trimester, but the authors point out that much of the research suffered from potentially serious methodological limitations, including lack of control for confounding factors and lack of data on adverse outcomes such as the presence of congenital malformations in aborted pregnancies [176]. 

Use of BZDs and HBRAs has, however, been shown to increase risk of low birth weight and premature birth. Women who use BZDs during pregnancy may have 6.8 times greater odds of preterm delivery, with risk being higher for women who use multiple medications and those who begin medication use later in pregnancy [177]. The timing of exposure may also impact outcomes. Earlier exposure may raise the risk for hypoglycemia, while later exposure to BZDs or HRBAs predicted both lower APGAR scores at 5 minutes post-birth, as well as a diagnosis of neonatal respiratory problems [91]. Benzodiazepine use is also positively associated with a greater likelihood of NICU admission [177]. A meta-analysis of clinical data from 1998 to 2010, which included a sample of over a million pregnancies, found that BZD exposure during first trimester did not significantly raise the risk of major malformations, including cardiac malformations [178]. However, studies may obscure effects because they often consider BZDs as a class rather than assessing risk by specific compound [176]. Diazepam may be the most well-studied of the BZDs with respect to use during pregnancy [179].

Zolpidem is an imidazopyridine with high selectivity and affinity for BZD receptor subtypes, and is a strong sedative with weak anxiolytic effects. Preclinical studies typically focus on diazepam and alprazolam in order to investigate the differential effects of drugs that act on the GABA system. There are few published studies that have been designed to specifically investigate the short- or long-term neurodevelopmental effects of gestational or neonatal zolpidem exposure. In a behavioral study comparing the effects of zolpidem, diazepam, and alprazolam in prenatally exposed rats, exposure to zolpidem did not cause significant behavioral effects in any of the behavioral tests, even though the doses given to the pregnant rats did produce sedative effects. The authors propose that this may be explained by receptor selectivity [180,181,182]. Similar results were found for a study of the study of adult rats exposed to picrotoxin and desipramine that were prenatally exposed to diazepam, alprazolam or zolpidem. Prenatal exposure to diazepam and alprazolam in these adults mediated the effects of later drug exposure on behavioral tests for exploratory behaviors and depression, and increased sensitivity to drug-induced seizures. Prenatal exposure to zolpidem was ineffective for all three tests. These data suggest that drugs that differentially act on GABA receptors may produce distinct behavioral outcomes [183]. 

The results of preclinical research suggest that BZD use is not teratogenic, but might induce long-term subtle developmental or behavioral changes. Research is limited, but the evidence so far does not suggest that fetal zolpidem exposure causes any short- or long-term adverse effects.

#### 3.5.2. Long-Term Developmental Outcomes: BZDs and HRBAs

The majority of early BZD studies used rat models of neonatal or postnatal exposure that involved drug treatment of pups during the first few postnatal weeks [184,185,186,187], which would be equivalent to the entire human neonatal period, from the third trimester until the first few weeks of life (the first week of life in the rodent roughly corresponds to the last trimester of human gestation). These studies did find evidence for subtle long-term changes in adolescent and adult rat behavior due to early BZD exposure that were drug-, dose- and time-dependent, and included short-term neurobehavioral effects on neonatal motor control, and long-term cognitive deficits and changes in social behaviors. For example, the startle response and some learning tasks were affected by prenatal diazepam, whereas submissive behavior was affected by neonatal lorazepam [175]. The long-term effects of postnatal diazepam treatment resulted in increased activity levels and delayed acquisition learning (without memory impairment), and increased behavioral response to novel stimuli. Results also showed metabolic decreases in brain regions mediating anxiety, which would be consistent with behaviors associated with anxiety and emotionality [188]. 

A more (relatively) recent rat study examined the influence of pre- or neonatal diazepam treatment on early neurodevelopment, and found that prenatal treatment resulted in a developmental delay for some neonatal reflexes (cliff aversion, forelimb placing, forelimb grasping and bar holding), but not others (righting and startle reflexes). Neonatal exposure to diazepam caused a neurodevelopmental delay only as measured by cliff aversion and startle reflex. Interestingly, this study found sex-differences in adult response to drug-induced seizures. Adult female rats exposed to diazepam as neonates showed a resistance to drug-induced convulsions, but males did not. These data suggest that prenatal exposure to diazepam induces long-lasting neurological and behavioral changes that may be sex-dependent [189]. The results of animal research suggest that fetal and/or neonatal exposure to BZDs can cause teratogenic effects and receptor-mediated changes in the brain that result in long-term neurodevelopmental effects that involve anxiety-related social behaviors. 

In the clinical research available in humans on the effects of hypnotic, non-benzodiazepine receptor agonists (i.e., “Z-drugs”–zolpidem, zopiclone, zaleplon), during pregnancy, results have shown that they do cross the placenta [190,191,192]. Subsequent research is limited, but has not found this class of drugs to be associated with teratogenic effects [91,92]. However, zolpidem use during pregnancy has been linked to adverse short-term pregnancy outcomes, including low birth weight, small for gestational age, and preterm and cesarean delivery [193]. Additionally, toxicology research does present this class of drugs as potentially hazardous in terms of risk of poisoning and adverse neuropsychiatric events that include parasomnia, hallucination, and amnesia. There have also been cases reported of women abusing zolpidem—in one case study exposure in the first trimester produced neural tube defects, suggesting that teratogenicity is possible at high enough doses [194].

A population-based study in Norway surveyed 45,266 mothers on their medication use during and after pregnancy. Mothers self-reported the use of anxiolytics/hypnotics in 0.8% of pregnancies, (most commonly, BZD anxiolytics and hypnotics such as zopiclone or zolpidem). The authors did not find any significant association between use of these medications and mother-rated language competence in the offspring at the age of 3 [195]. A review of the literature on longer-term outcomes produced mixed results, with some evidence for a link between BZD exposure and impaired motor or mental development, and some evidence for no link between exposure and significant impairment. Overall, given the variety in outcome measures, the authors were unable to translate the results of their research into clinical recommendations and concluded that more studies are needed [190].

### 3.6. Conclusions—Benzodiazepine Use during Pregnancy

The greatest risk to the fetus appears to be when these medications are administered two to eight weeks after conception [179]. Diazepam and chlordiazepoxide (both long-acting compounds) appear to be the safer choices for use in the first trimester of pregnancy [179,180]. In contrast, alprazolam, a short-acting compound, may a greater risk of negative outcomes such as congenital anomalies such as cleft lip, microcephaly, and umbilical hernia [179]. It should be noted that while monotherapy may generally be considered safe, combining BZDs with SSRI antidepressants has been associated with a greater risk of congenital heart problems [115]. It may be advisable for BZD use, particularly at high doses, to be avoided, especially during the final trimester as exposure in late pregnancy can result in neonatal adaptation, or “floppy infant syndrome” also seen with exposure to other agents. This may be of particular concern with longer-acting compounds such as diazepam and nitrazepam [196]. 

A methodological concern in this area is a frequent lack of inclusion of a comparison group of fetuses exposed to maternal anxiety symptoms but not medication, potentially creating a confound [157]. However, Ban and colleagues [92] addressed this by comparing infants who had first trimester exposure to benzodiazepines to infants whose mothers who had anxiety/depression but were not taking medication. Exposure to medication did not significantly raise the risk of major congenital anomalies. Overall, research on long-term outcomes is limited, and there are often methodological concerns [197]. 

Pregnancy is often a time when a woman may experience sleep disturbances, even if she has never had them before. Chronic insomnia is often worsened by pregnancy, as are other disorders that disrupt sleep, such as restless leg syndrome and sleep apnea. The evidence suggests that drug treatment for sleep disturbances during pregnancy may have harmful effects on fetal and neonatal outcomes. Similar to any other health problem, left untreated, severe sleep disorders can also have an adverse effect on mother and baby. Similar to any other drug treatment, the health risks of inability to sleep must be balanced against the potential for drugs to cause adverse pregnancy outcomes that include prematurity, low birth weight, and a higher chance for a cesarean delivery. Alternative drug-free treatments may be indicated, such as cognitive behavioral therapy, which has shown to be effective [197], so should be used as a first-line treatment for sleep problems during pregnancy.

### 3.7. Beta-Blockers (BBs)

#### 3.7.1. Fetal and Neonatal Effects

The use of BBs during pregnancy has typically been studied in the context of women who are using these medications in the treatment of hypertension or cardiovascular disease. Therefore, caution should be exercised in generalizing these results to pregnant women with anxiety, as most studies utilize a control group of women who have untreated high blood pressure. Given that limited research examines BB use for anxiety, it is difficult to separate the impact of the underlying condition on the outcomes of interest. For example, an association with being born small for gestational age was found for beta-blocker use among pregnant women with pre-existing hypertension, but not for those taking the medication for gestational hypertension [198]. A review of animal research [199] on exposure to atenolol found evidence of embrotoxicity in rats and rabbits when given at doses higher than the maximum human dose. Exposure was linked to lower birth weight in rats and rabbits, and rats exposed to atenolol showed bradycardia in the first day of life. 

Beta blockers have not been associated with teratogenic effects [200]. Use of BBs during pregnancy is associated with fetal growth restriction (FGR) when used by women with heart disease [201] and low birth weight when used in the early pregnancy for hypertension [202,203]. A meta-analysis of randomized trials for pregnancy hypertension notes that the impact of BBs on size for gestational age have been inconsistent, but appear to slightly increase the risk of being born small for gestational age when used to treat mild chronic hypertension [204]. 

Differences within drug class have emerged, and could possibly explain the inconsistent results. Among BBs, use of atenolol, propranolol, and metoprolol were associated with instances of FGR, while bisoprolol was not [205]. Atenolol is one of the more well-studied drugs in this class, and its negative impact on birth weight has been noted. Use of atenolol in early pregnancy (from conception, or in the first trimester) is associated with a greater risk of low birth weight as compared to use initiation of use later in pregnancy [206]. Because of this, providers may be cautioned against using atenolol during pregnancy [204]. Labetalol may be a safer choice [200]. Atenolol appears to lead to decrease umbilical venous blood flow after one week of treatment and lower placental weights [207] which could perhaps contribute to the lower birth weights. Longer treatment duration with atenolol or other BBs is associated with greater risk of FGR [205,208]. 

Cardiovascular teratogenic effects of beta blocker use were observed in one study [209], but not another [210]. Concerns have been raised about the potential negative impact of BBs on fetal hemodynamics [207]. However, a systematic review did not find strong evidence of an impact of antihypertensive drugs such as labetolol on fetal heart rate [211]. Another study showed that BB use in the third trimester raised the risk of hypoglycemia in the infant, but did not raise the risk of congenital anomalies [212].

#### 3.7.2. Long-Term Developmental Effects: BBs

Little research exists which examines the long-term impact of fetal exposure to BBs, in either animals or humans. In rats, prenatal exposure to propranolol was found to impair spatial maze problem solving in female rats when they were raised in impoverished conditions [213]. A randomized trial in humans which assessed use in the third trimester of atenolol found no adverse effects through the first year of life, relative to placebo [214].

### 3.8. Mood Stabilizers: Lithium, Anti-Psychotics and Anti-Epileptics

#### 3.8.1. Fetal and Neonatal Effects

##### Lithium

Lithium (Li) has long been the first-line treatment for bipolar disorder. As early as the 1970s, Li was identified as a teratogenic agent in human infants responsible for a congenital malformation known as Epstein’s anomaly, which is a malformation of the structure and function of the heart valves, and the presence of an atrial septal defect (a hole in the wall separating the atria). Subsequently, most of the early preclinical studies were focused on fetal teratogenicity—specifically heart, liver, and kidney malformations. There is evidence from early animal studies that Li is embryotoxic and teratogenetic [215,216], but the doses have been reported as too high to be clinically relevant [217]. Subsequent data suggested that any teratogenic risk is smaller than previously thought, but while the overall risk is low, it is still higher in infants exposed to Li [218,219,220]. A systematic review of information published from 1969 to 2005 on the risk of major congenital malformations with prenatal exposure to Li concluded that lithium should not be considered a “major” human teratogen, and that Li should be administered to pregnant women for treatment of maternal BPD [221]. Concerns are not limited to exposure during the first trimester. Third-trimester exposure to Li late in pregnancy, or neonatal exposure through breastfeeding, has also caused adverse effects, specifically a neonatal adaptation syndrome, described in early case studies as “floppy baby syndrome” that includes lethargy, hypotonicity and cyanosis [222], and more recently has been observed to also involve symptoms such as muscle twitching, respiratory problems, feeding difficulties, cardiac arrhythmias, and poor reflexes [223,224,225]. One study also found that lithium-exposed infants had significantly higher birth weights [219]. 

Interestingly, Li was still investigated then [226], and is now [227], as a potential protective agent against fetal alcohol syndrome; those studies focused on the effects of Li on physiological development of the liver and kidney in neonates. Even in those studies, however, Li was described as a potentially neurotoxic agent [226]. Recently, research using animal models has suggested that Li may be neuroprotective against an almost impossibly long list of diseases and disorders, including Alzheimer disease [228], Huntington’s disease [229], hypoxic-ischemic brain injury [230,231], and neurodevelopmental disorders such as fragile X [232], Down syndrome [233,234], and autism [235]. The results of these studies may be extended to suggest a neuroprotective role of Li against short- and long-term adverse effects of maternal BPD, but this possibility must be weighed against the long history of results of studies showing the potential for teratogenic effects in human infants prenatally exposed to Li. 

##### Antipsychotics

In a recent comparative study, valnoctamide, an anxiolytic drug developed in the 1960s that has re-emerged as a potential candidate for epilepsy and BPD, was compared to risperidone and olanzapine in a mouse model designed to determine teratogenicity. The focus was on adverse pregnancy outcomes in the mouse dam, including failures of implantations, resorptions, fetal loss and gross anatomical malformations. The results showed that olanzapine and risperidone are both teratogens and fetotoxic, with olanzapine as the most teratogenic. Rispiridone induced anatomical (e.g., cleft palate) and skeletal abnormalities at higher doses. Olanzapine, however, induced maternal toxicity, embryo resorption, reduced fetal weight, structural malformations and delayed growth. Valnoctamide did not show any teratogenic effects, even at higher doses. However, in this study there were skeletal malformations in one of the valnoctamide treatment groups that the authors concluded indicated developmental delay rather than major structural malformations [236].

Atypical antipsychotic drugs such as second generation quetiapine and third generation aripiprazole have been suggested as a treatment option for BPD. In animal studies designed to investigate the consequences of gestational exposure to quetiapine and aripiprazole on maternal and fetal outcomes, fetal and postnatal body weight gain was significantly reduced in exposed rats [237,238]. This reduction in growth and development was long-term and suggests that atypical antipsychotics may not be safe for use during pregnancy.

Some studies of the pregnancy and neonatal outcomes of maternal antipsychotic use have shown that these drugs are associated with congenital malformations, preterm birth, and abnormal fetal growth [239,240,241,242]. Others have not found a connection between fetal growth, with the exception of an increased risk of macrocephaly associated with clozapine and olanzapine [243]. In a subsequent study, Bodén, et al. [26] reported that both untreated and treated women had increased risks of cesarean/instrument assisted birth, induction of labor, and preterm birth. Growth measures, such as weight, length and head circumference were not significantly different among treated women. It was concluded that women with BPD were at an increased risk for adverse outcomes that included premature birth, regardless of drug treatment. A retrospective comparative cohort study found that women treated with antipsychotics are at a higher risk for birth by cesarean section and adverse outcomes that included premature birth and low birth weight. These risks were significantly reduced when adjusted for health and lifestyle factors. The overall conclusion was that there is limited risk associated with exposure to typical and atypical antipsychotics [244]. Coughlin et al. [245], however, conducted a meta-analysis of studies designed to assess pregnancy and neonatal outcomes. They concluded that the analysis was limited because there is a small amount of well-controlled studies, but that antipsychotic exposure was associated with an increased risk of major malformations, heart defects, small for gestational age, and low birth weight. 

##### Antiepileptics (AEDs)

Animal studies have provided ample evidence that the AEDs are teratogenic and neurotoxic, and can result in craniofacial structural abnormalities, altered neurodevelopment, and long-term behavioral and cognitive deficits [246,247]. Some AEDs affect neuronal growth and survival by causing widespread neuroapoptosis [248,249,250], and by inhibiting neurogenesis [251]. These effects are dose-dependent, and careful consideration has been given to study therapeutically relevant dose levels. Importantly, the effects are observed with relatively brief exposure periods shorter than what is likely to occur if treatment is continued throughout pregnancy. 

A number of AEDs have been studied and used for treatment of bipolar disorder, but only valproate, carbamazepine, and lamotrigine have FDA approval for use in BPD. In humans, valproate and carbamazepine present a more potent teratogenic risk than lithium. Valproate exposure was quickly associated with birth defects, which include neural tube defects (spina bifida, anencephaly), craniofacial abnormalities, intrauterine growth retardation, microcephaly, and heart defects. The condition, sometimes referred to as valproate syndrome, is phenotypically characterized by facial hypoplasia, a short nose with upturned nostrils, and a long upper lip [252]. These teratogenic effects, however, have been associated with both carbamazepine and valproate exposure [253], and risk is higher with combined therapy than with monotherapy [254], so the combination of valproate and carbamazepine is not recommended. A recent analysis of umbilical cord and maternal serum levels revealed that there is a significant correlation between maternal and umbilical cord serum valproate levels, and a negative correlation between serum levels and birth length and weight, such that high serum levels were associated with decreased birth size, and this was independent of dose [255]. The findings of other recent studies confirm the results of previous research—valproate exposure increases the risk of multiple congenital anomalies that include spina bifida, atrial septal defect, cleft palate, hypospadias; polydactyly, and craniosynostosis [256]. Malformation rates increase according to dose at time of conception with most AEDs, including phenobarbital, valproate, carbamazepine, and lamotrigine [257]. These agents are also associated with a neonatal adaptation syndrome characterized by irritability, feeding problems, abnormalities in muscle tone, liver toxicity, clotting disorders, and hypoglycemia.

Lamotrigine, the most recently approved drug for maintenance treatment of BPD, appears to be associated with a lower rate of malformations compared to valproate, and is also now considered to be a first-line treatment for epilepsy. However, this drug is also not without risk. In a study that compared medicated to unmedicated women with epilepsy, the incidence of miscarriage or stillbirth was significantly higher in anticonvulsant-treated women with epilepsy [258]. Data from the North-American Anti-Epileptic Drug Registry indicates a higher risk for oral cleft in infants exposed to lamotrigine. However, other registries do not indicate a significant increase in risk. 

Prenatal exposure to some AEDs is associated with a greater risk for organ malformation, and is dependent upon dose and time of exposure during gestation. Valproate is by far the most dangerous drug at all stages of fetal development; higher maternal serum anticonvulsant levels and exposure to more than one anticonvulsant further increases risk. A review of the teratogenic potential of common bipolar agents found that valproate is associated with the highest rate of major congenital malformations (6.2%–16%). The relative risk of neural tube defects with valproate and carbamazepine is reported as approximately 1%–5% and 0.5%–1%, respectively [259]. Because of the risk of neural tube defects, valproate is not recommended for women during their reproductive years, because the risk for birth defects is so high in very early pregnancy, before many women realize they are pregnant. As would be expected, the rate of congenital malformations is higher in fetuses exposed to drug combinations compared to monotherapy. A summary of the drugs approved by the FDA to treat bipolar and psychiatric disorders is provided in Table 3.

#### 3.8.2. Long-Term Developmental Effects 

##### Lithium

Studies on long-term behavioral teratogenicity are inconclusive due to a lack of research designed to evaluate neurodevelopmental outcomes beyond the neonatal period. Because of the unique characteristic of mood swings in BPD, there are also no universally accepted animal models of BPD. Therefore, animal data on long-term behavioral effects are limited. In 2006, Youngs et al. [260] investigated the behavioral teratogenicity of neonatal exposure to Li in rat pups. The results suggested that brain development is altered and was reflected in persistent anxiety-like behavior in adult rats. In a study of gestational Li exposure, Abu-Taweel [261] observed delays in developmental milestones and sensorimotor and activity/locomotor deficits. 

Until recently, only one study, decades old, had investigated children exposed to Li in utero, and reported that the children did not differ behaviorally from their unexposed siblings [93]. In 2012, a qualitative study of 15 prenatally exposed children assessed growth, neurological, cognitive and behavioral development at 3–15 years of age. The researchers identified one child who had minor neurological problems that had no clinical implications. Cognitive test scores were within the normal range, but most children had lower performance IQ scores. All other developmental outcomes were normal [262]. In a systematic review of the use of mood stabilizers during pregnancy, Galbally et al. [263] concluded that a lack of data made it impossible to determine the effects of Li exposure on long-term childhood behavioral outcomes. 

##### Antipsychotics

Animal studies that assess long-term neurobehavioral effects of early drug exposure typically assess behaviors using activity/locomotor, social, and cognitive tests. Singh et al. [264] showed that the long-term effects of the typical antipsychotic haloperidol was dependent on the gestational day of exposure. Early gestational exposure on day 9 (GD9) caused decreased rearing and locomotor behavior, whereas exposure on GD14 increased locomotor behavior in adult rats. Both groups of rats showed anxiety- and depression-like behaviors compared to controls, with an increased effect seen in rats exposed on GD14. In a study of more continuous gestational exposure, adult rats exposed to haloperidol from GD 12 to 20 also showed increased anxiety-like behavior compared to untreated controls [265]. Results of a rat study that used gestational exposure paradigms found that typical (i.e., haloperidol) and atypical antipsychotics (olanzapine, quetiapine, risperidone) produced differential effects on learning and memory, whereas haloperidol, risperidone and quetiapine impaired learning, and haloperidol and risperidone impaired memory [266]. More recently, Zuo et al. [267] compared sulpiride (an antipsychotic not approved for us in the U.S.) and risperidone in a rat gestational exposure model (GD 6 to 18). Fetal exposure to sulpiride produced decreased exploratory behavior, and impaired performance in a cued learning task, but did not cause impaired memory in adult rats. Risperidone exposure did not have any effect on exploratory behaviors or learning and memory performance. This difference was proposed to reveal how the mechanism of action of two drugs in the same class, sulpiride (selective dopamine D2 receptor blockade) and risperidone (dopamine D2 and serotonin 5HT2 receptor blockade), can have different neurodevelopmental and long-term behavioral consequences [267].

There are few studies in humans that have systematically assessed the long-term neurobehavioral outcome of exposure to antipsychotics during pregnancy. Most studies focus on teratogenic effects, and pregnancy/neonatal outcomes. Few studies evaluate long-term behavioral teratogenicity in children exposed to antipsychotics in utero. In 2013, Peng et al. [268] evaluated the effects of atypical antipsychotics on infant development and reported that fetal exposure to atypical antipsychotic drugs was associated with delays in cognitive, motor, socio-emotional behaviors at 2 months of age, but these effects were no longer seen at 12 months. In a study of the effects of fetal exposure to clozapine, risperidone, olanzapine, or quetiapine, clozapine was associated with delayed development in infancy (2–6 months of age), but there were no differences among the drug treatments for infant cognitive, language, motor and socioemotional skills from 2 to 12 months of age [269]. 

##### Antiepileptics (AEDs)

Of all of the anticonvulsant drugs, valproate has the most definitive evidence for teratogenic and long-term adverse neurobehavioral effects. All animal studies that have investigated the anatomical and behavioral effects of in utero valproate exposure show some level of adverse outcome. Valproate was quickly identified as a drug that causes social deficits specifically; results of those studies show that valproate exposure in both rats and mice leads to autistic-like behaviors that includes deficits in social and motor behavior [270]. This effect of valproate is so robust, in fact, that valproate exposed mice are considered an animal model for preclinical studies on autism.

Most of the major retrospective studies compare valproate to controls or to other anticonvulsants, such as carbamazepine and lamotrigine. Valproate is the only anticonvulsant drug that is FDA-approved for the treatment of mania. A major conclusion of the authors of the most recent reviews on the long-term effects of fetal exposure to anticonvulsants [271,272] is that there is convincing evidence that valproate-exposed children have a lowered IQ, and motor, social, and communication deficits that are significant enough to have an effect on educational and occupational outcomes. This effect for valproate is dose-dependent [273,274]. 

For the other anticonvulsants that may be used to treat BPD, such as carbamazepine and lamotrigine [275,276], the studies that have been conducted have mostly compared antiepileptic treatment groups, without a healthy control group, and have found that valproate use carries the highest risk. There have been studies that have found a connection between carbamazepine exposure and increased risk for fetal malformations, but compared to valproate, it is not a particularly potent teratogen [277,278]. Other drugs, such as lamotrigine and levetiracetam, are commonly evaluated relative to valproate, but ethical and practical considerations make it difficult to conduct studies with adequate comparison groups or even comparable methodology. More research is needed on the newer AEDs in order to come to a firm conclusion about long-term effects. 

### 3.9. Conclusions: Mood Stabilizer Use during Pregnancy

Preclinical research using animal models provides converging evidence for the adverse pregnancy and neonatal outcomes experienced by women who are under drug treatment for mood disorders while pregnant. The results of histological work reveal the potential underlying neuropathological mechanisms for the neurodevelopmental problems observed in humans. For example, animal studies show that psychoactive drugs may affect neurodevelopment by either inhibiting normal neuroapoptosis (i.e., lithium), or causing neurotoxic cell death in combination with inhibited cell growth (i.e., anticonvulsants and antipsychotics). This may account for the gross abnormalities in brain size and weight observed in animal models, and would be consistent with the neurological deficits observed in human infants and children who are developmentally delayed or exhibit learning and intellectual disabilities. The results of animal research also support the findings in humans that anticonvulsants and antipsychotics may cause adverse pregnancy outcomes that could have long-term consequences for childhood development.

The results of prospective and case-control studies showed that Li exposure increases the risk of Ebstein’s anomaly, although more recent studies suggest that the risk may be lower than previously reported. The potential risk of neural tube defects cannot be ruled out. Despite these concerns, lithium remains the first-choice agent for treating BPD in pregnancy. However, women who need Li treatment during pregnancy require careful and strict clinical surveillance. Preconception folate supplementation is advised to reduce the risk of neural tube defects, and should be combined with a careful plan for prenatal and neonatal care, including a labor and delivery clinical plan in place, to ensure proper neonatal care because of the higher risk for complications [279].

Major prescribing changes have taken place over the years as new drugs are developed and tested. It is important to remember, however, that a newer drug or treatment regimen does not guarantee safety. For example, the decrease in the use of lithium and the increase in the use of anticonvulsants for BPD is not consistent with recommendations from international guidelines. Clinicians and researchers from around the globe have written extensively about the use of mood stabilizers during pregnancy, which include comprehensive reviews of lithium, antipsychotics, and off-label use of antiepileptic drugs [263,279,280]. Several perinatal complications, mostly falling under the category of what is known as neonatal adaptation syndrome, may occur in the case of drug exposure during late pregnancy. In 2011, the U.S. FDA issued a safety announcement for the entire class of antipsychotic drugs, specifically citing extrapyramidal signs (EPS) that involve problems with neonatal muscle tone, tremor, sleep, breathing, and feeding [281]. Major teratogenic effects are consistently reported for valproate and carbamazepine. There is limited evidence for lamotrigine as one of the safest antiepileptic drugs to be used for BPD in pregnancy. Preliminary studies also report no increased risk for major congenital malformations associated with gabapentin or oxcarbazepine exposure. 

All mood stabilizers and antipsychotics, however, appear to carry some form of increased risk to the fetus and neonate. Short-term effects include increased risk of congenital malformations and poor perinatal outcomes. Studies that examined longer-term neurodevelopmental consequences found poorer outcomes for those children exposed to sodium valproate or polytherapy in pregnancy than for those exposed to other individual antipsychotics. The data on longer-term child development outcomes with Li exposure are too limited to draw any conclusions. We must also keep in mind that most human studies do not take into account differences between mono- and combination therapy. 

## 4. Limitations of the Study

In this paper, we review the available studies used by clinicians, researchers, patients, and oversight agencies like the FDA to determine the risks of psychoactive drug exposure during pregnancy. Because a major limitation of preclinical animal models is that they do not involve treatment of animals in a depressed, anxious, or other psychiatric state, the translational relevance of the findings of these studies to humans is questionable at best. It could be argued that such preclinical evidence cannot, and perhaps should not, be used to assess drug safety. Therefore, a major limitation to this research is that we are comparing available preclinical research to clinical research, but cannot reliably state that there is strong clinical relevance.

## 5. Conclusions

### 5.1. Scope of Problem

Potentially any drug used during pregnancy could cause adverse effects for the fetus, as most, if not all, therapeutic substances freely cross the placenta. Women, pregnant women, and children are understudied classes of the population who may differentially experience adverse effects of therapeutic drugs. For most mental illnesses, the risk of going without treatment equals or even exceeds that associated with psychoactive drugs. The task for preclinical researchers is to determine which drugs are the safest for use during pregnancy, and the task for clinicians is to responsibly prescribe them. Recent increases in the rates of diagnosis of psychiatric disorders mean that it is likely that the prevalence of pregnant women being treated with therapeutic drugs for these disorders will continue to rise. 

### 5.2. Information Sources

The advice most often provided to women who have questions about whether a drug is safe during pregnancy is to “talk to your doctor.” What this review reveals is that unfortunately, in most cases, there is not enough evidence for the most well-informed doctor to be able to say with certainty that any drug is 100% safe, or even estimate the risks. A 2010 survey of obstetricians and gynecologists found that the two most commonly reported barriers to counseling pregnant women on medication use were lack of sufficient information about medication safety for the fetus and difficulty interpreting the information that was available. These physicians also reported that they most frequently consulted print materials as their source of information (such as the Physicians’ Desk Reference and other books). Only 31% reported most frequently turning to online resources, which arguably may be more up-to-date than printed books [282]. Today’s pregnant women, concerned about the effects of drugs on their developing child, frequently use sources other than their physician, such as the internet, for information. Research has shown, however, that the use of internet sources may result in women relying on incorrect and wildly inconsistent information [283,284]. 

Fortunately, there are now some very reliable sources that are easily accessible online, such as on the government sites for the FDA and CDC, and the Organization of Teratology Specialists (OTIS), a professional scientific society that provides the Mother-to-Baby service dedicated to providing the best and most up-to-date information on medication use during pregnancy [285].

The complexity of maternal-fetal medicine and the difficulty in determining and communicating the risk vs. benefit of therapeutic drug use has recently been the focus of the Food and Drug Administration (FDA). The FDA recognizes that women of childbearing age may have to begin or continue to take medications while pregnant, and drug labeling laws should reflect that fact. The result is a new labeling rule that drug manufacturers must follow in providing information about the risks of a drug during reproductive age, pregnancy, and lactation. There are no psychiatric drugs that have been specifically tested and approved by the FDA for use during pregnancy. Citing a need to provide better information to clinicians and patients regarding the safety of drugs during reproduction and pregnancy, the FDA enacted a new policy, the Pregnancy and Lactation Labeling Rule (PLLR), that eliminates the pregnancy letter category labeling system (A, B, C, D, and X) and requires changes to both the content of, and format for, presentation of information in prescription drug labeling. The goal is to assist health care providers, pregnant women, and nursing mothers who need to take medications in their assessment of benefit versus risk so that they are better able to make informed and educated decisions. An important additional change is that the PLLR also requires the label to be updated when new information becomes available. This rule applies to all drugs approved since 2001, and requires that drug companies provide more specific information about human and animal data, adverse reactions, and dose adjustments required during pregnancy and post-partum periods. This new labeling rule is designed to provide better information, but cannot reliably do so without adequate data to support prescribing recommendations. Because the psychoactive agents discussed in this paper were developed and approved long before the new labeling rule applies, drug manufacturers are not required to comply with the PLLR decision, so these drugs may have lost their letter designation, and no new information is required to be provided.

### 5.3. Status of the Literature

Over the years, human studies have steadily improved with respect to clinical research study design and methodology in order to better define the potential impact of maternal illnesses and drug exposures. Animal studies are plagued with the usual problems concerning methodological inconsistencies across studies, questions about relevance of drug dosage and exposure, and whether the overall results translate to humans. Ideally, in order to determine the true impact of pharmaceuticals during pregnancy, preclinical animal models would limit the exposure to the drug according to the timing of the expression of the neurotransmitters and receptors, and the development of neural circuitry directly affected by different classes of psychoactive drugs. The fact that much of the previous research in animals may have not considered the exact timing of these neural markers as part of the methodology regarding age of exposure makes it even more difficult for researchers to adequately determine the true potential impact of drug exposure during pregnancy. Additionally, a major limitation of most, if not all, animal models used to test prenatal drug exposure is that the drugs are administered to “healthy” animals. This further complicates the issue as to whether results from these experiments translate to the human condition of having a disorder and undergoing treatment. Using non-affected animal models to determine risk may therefore be inappropriate. The results of animal studies, are, however, mostly convergent with the results of clinical research. Human studies also have a unique set of problems, mostly involving control issues. Specifically, they often lack proper healthy or non-drug-exposed, affected control groups of pregnant mothers. For case-control or cohort studies, there are a large number of confounding psychosocial factors that may influence the outcome measures, such as comorbid illness, poly drug exposure, and environmental lifestyle and health care factors, especially prenatal care. Similarly, the literature on the potential adverse effects of untreated illness may be equally confounded. This may explain repeated findings, across studies, disorders, and drug exposures that overlap, such as preterm delivery, low birth weight, and other short-term neonatal complications. Women with a mental illness diagnosis also may engage in other high-risk, even dangerous, behaviors that adversely affect their health, which may preclude the suspension of drug treatment during pregnancy. Lastly, much of the literature has focused on short-term birth outcomes rather than longer-term health and behavioral outcomes, so less is known about whether fetal exposure to drugs causes permanent changes in social and cognitive abilities that last into adulthood.

## 6. Future Directions

More research is needed on both the safety and efficacy of pharmacotherapy during pregnancy. Specifically, more carefully designed preclinical studies are needed to clarify the exact risks for different medications in terms of single- or poly-drug exposure, and that assess neurodevelopmental and behavioral effects. More clinical research investigating psychoactive drugs used to treat mental illnesses and disorders in pregnant women is desperately needed. There is, in fact, a parallel to this research problem in the field of pediatric and neonatal medicine in the study of the effects of early exposure to sedatives and anesthetics on the developing brain. The discovery from animal studies that anesthetic and sedative drugs may by neurotoxic during the brain growth spurt period led to a major movement to conduct extensive retrospective studies investigating whether or not early exposure to sedative and anesthetics is associated with childhood developmental delays and behavioral disorders. This retrospective research led to a call for prospective studies that carefully monitor exposure and evaluate specific (rather than global) neurobehavioral outcomes. This field of research that investigates the effects of psychoactive drugs on the developing brain requires the same level of attention and rigorous research devoted to assessing how drug exposure during a critical period of brain development may have permanent effects with consequences that last into adulthood. This would provide better information for doctors and their patients, and better inform parents and pediatricians in the evaluation and assessment of children who may be predisposed to developmental or behavioral problems due to fetal drug exposure.

Alternatives to drug therapy should also be studied and considered, including more intense psychotherapy or cognitive behavioral therapy to replace or reduce the need for medications to treat women with mental illnesses and disorders—especially for mild cases. In the meantime, information about the effects of drugs on fetal and neonatal outcomes needs to be more fully and freely disseminated and put to good use in the field of women’s health. It must be recognized that pregnant women with significant mental health illness and disorders need more than the typical level of prenatal care, and it must begin before a pregnancy is planned in order to allow for the best outcome for mother and baby. Where the health of our most vulnerable obstetric patients is concerned, we can and should do better. 

## 7. Summary Points

- While the FDA seeks to improve information regarding risk of medication use during pregnancy, there are no clear guidelines regarding the use of ADs during pregnancy.
- Any potential drug-exposure risks to the child must be balanced against the equally risky effects of untreated mental illness or disorder in the mother, especially for serious mood disorders such as major depression and bipolar disorder.
- The results showing adverse effects of maternal mental illness on the child suggest that adequate pharmacotherapy may need to be maintained before, during, and after pregnancy.
- Any additional pregnancy complications would require even closer monitoring of potential drug-related effects due to multiple risk factors related to physiological changes during pregnancy, including changes in circulating levels of hormones, cardiovascular changes, increase in glomerular filtration, postural hypotension, anemia, and increased metabolic rate, and insulin resistance. Management of any disorder during pregnancy should include monitoring of serum drug levels.
- Decisions regarding both medication use and discontinuation during pregnancy should be made carefully, taking full psychiatric history into account, including severity, chronicity, and co-morbidity of the mental illness, disorder, or condition.
- Regarding teratogenicity, it is important to remember that any risk should be compared to the baseline risk of major congenital malformation, which is approximately 2%–4% in the U.S. The same is true for risk of an autism spectrum disorder.
- More rigorous preclinical and clinical retrospective and prospective research is needed to provide better evidence and better inform clinical practice.

## Figures and Tables

**Table 1:** Pregnancy risks and outcomes associated with untreated maternal disorder.

MATERNAL DISORDER	PREGNANCY RISKS AND OUTCOMES
**DEPRESSION DISORDERS** Major Depression Persistent Depression Disorder Minor Depression	inadequate maternal weight gain [16] substance abuse [17] pre-eclampsia; preterm birth; low birth weight [18,19,20,21,22] fetal distress [23] increased risk of cesarean birth; increased risk of neonatal intensive care unit( NICU) admission [24]
**BIPOLAR DISORDERS** Bipolar I and II Bipolar NOS Cyclothymia	low birthweight, size at birth, preterm birth [25] increased risk of cesarean birth, small head circumference, hypoglycemia [26] increased risk for long-term neurocognitive, behavioral and social deficits [27,28,29] high postpartum risk for first-onset and recurrent bipolar episodes [30,31,32,33,34] and hospitalization [30,31,35,36] substance use, poor prenatal care, maternal suicide [37,38,39]
**ANXIETY DISORDERS** Generalized Anxiety Disorder (GAD) Panic Disorders Social Anxiety Disorder Specific Phobias	increased risk for preterm birth, small for gestational age [39,40,41] spontaneous abortion [42] pre-eclampsia, deceased head circumference [43], low birth weight [44] excessive infant crying [45] long-term childhood behavioral disorders, anxiety [46] altered maternal/fetal cortisol levels [47]
**OBSESSIVE COMPULSIVE DISORDER**	low birth weight, preterm birth [48]
**SLEEP DISORDERS** Insomnia	comorbid with mood/anxiety disorders postpartum depression

**Table 2:** Overall prevalence (U.S. data), average age of onset, prevalence rates, and risk experienced by pregnant women.

Maternal Disorder	Prevalence	Age of Onset	Pregnancy Prevalence	Pregnancy Risks and Outcomes
**Depression** Major Persistent Minor	~15 million (6.7% of population)	32.5 years	4–25% [66,67,68] Rates of are equal for pregnant and non-pregnant women [8] Most prevalent maternal disorder	inadequate maternal weight gain [16] substance abuse [17] pre-eclampsia; preterm birth; low birth weight [18,19,69] fetal distress [20] increased risk of cesarean birth; increased risk of NICU admission [21,70]
**Bipolar Disorders** Bipolar I and II Bipolar NOS Cyclothymia	3–7% [7]	25 years	25–30% of pregnant women experience episodes [22,42]	low birthweight, size at birth, preterm birth [25,26] risk of cesarean birth, small head circumference, hypoglycemia [26] risk for long-term neurocognitive, behavioral and social deficits [27,28,29] high postpartum risk for first-onset, recurrent episodes [30,31,32,33,34] and hospitalization [30,31,35,36] substance use, poor prenatal care, maternal suicide [37,38,39]
**Anxiety Disorders** Generalized anxiety disorder (GAD) Panic Social Phobia	2.7% Most prevalent disorders in U.S. [7]	GAD: 30y Panic: 24years Social: 8–15 years Specific: 7–11 years [71]	Any disorder: 13% GAD: 1.3% Panic: 2.2% Social: 1.8% Phobia: 9.2% [72]	increased risk for preterm birth, small for gestational age [40,41,44] spontaneous abortion [42] pre-eclampsia, deceased head circumference [43], low birth weight [44] excessive infant crying [45] long-term childhood behavioral disorders, anxiety [46] altered maternal/fetal cortisol levels [47]
**OCD**	2.07% [57]	19.5 [34]	1.08% [57]	low birth weight, preterm birth [48]
**Sleep Disorders** Insomnias	24%–48% [67]	Any age beginning in childhood [73]	66%–94% [74]	comorbid with mood/anxiety disorders and associated adverse effects postpartum depression pre-eclampsia, gestational diabetes [73,74]

**Table 3:** Drugs approved by the FDA to treat depression, anxiety and sleep disorders, according to class of drug, the trade name used in the U.S. market, and the FDA pregnancy category assigned to each. The drug label information regarding pregnancy risk, as provided by the drug manufacturer, is summarized for the animal and human data, which often involves experiments using the maximum recommended human dose (MRHD).

DRUG CLASS and NAME	TRADE NAME	CURRENT DRUG LABEL INFORMATION *^b^*
**ANTIDEPRESSANTS**		
Tri- and *Tetra-cyclics (TCAs)		
Amitriptyline	Elavil	Few teratogenic effects are reported, except at doses of amitriptyline which far exceed the MRHD. Results of animal research on desipramime, nortriptyline, and imipramine are described as “inconclusive.” At doses >MRHD, increased pup mortality and low body weight were reported for amoxapine and doxepin. Trimipramine exposure at 20X MRHD caused an increased risk of major abnormalities. There are no adequate and well-controlled studies in pregnant women. Adverse events in humans (central nervous systemeffects, limb deformities, developmental delays) have been reported for amitriptyline. Neonatal withdrawal and anticholinergic symptoms have been observed. The kinetics of this drug change during pregnancy, serum levels should be monitored and the dose should be adjusted if needed.
Amoxapine	Asendin	
Desipramine	Norpramin	
Doxepin	Silenor	
Nortriptyline	Aventyl, Pamelor	
Protriptyline	Vivactil	
Trimipramine	Surmontil	
*Mirtazapine	Remeron	
*Maprotiline	Ludiomil	
**Monoamine Oxidase Inhibitors (MAOIs)**		
Phenelzine	Nardil	Phenelzine may increase fetal/pup mortality in rats. There is little information on the effects of exposure to tranylcypromine or isocarboxazid in animals. Exposure to selegiline at many times the MRHD increased the risk for major malformations (delayed ossification) and decreased fetal weight. There are no adequate and well controlled studies in pregnant women.
Tranylcypromine	Parnate	
Isocarboxazid	Marplan	
Selegiline	Eldepryl, Zeladar	
Serotonin Reuptake Inhibitors (SRIs) and *Serotonin-norepinephrine reuptake inhibitors (SNRIs)		
*General FDA warning: A study of women with history of major depression who were euthymic at the beginning of pregnancy showed women who discontinued AD medication during pregnancy were more likely to experience a relapse than women who continued medication use. Neonates exposed late in the 3rd trimester have developed complications requiring prolonged hospitalization, respiratory support, and tube feeding. Reported clinical findings include: respiratory distress, cyanosis, apnea, seizures, temperature instability, feeding difficulty, vomiting, hypoglycemia, hypo-/hypertonia, hyperreflexia, tremor, jitteriness, irritability, and constant crying. These features may be a direct toxic effect or a withdrawal syndrome. In some cases, the clinical outcome is consistent with serotonin syndrome.*		
Citalopram	Celexa	Animal studies did not suggest teratogenic effects for sertraline or escitalopram, and only at toxic doses for citalopram. At doses >MRHD, increased risk of skeletal abnormalities and decreased fetal growth/survival. There are no adequate and well-controlled studies in pregnant women. First trimester fluoxetine use is associated with increased risk of cardiovascular malformations; paroxetine is linked to cardiac malformations (ventricular septal and valve defects). Consideration should be given to either discontinuing paroxetine use or switching to another antidepressant.
Escitalopram	Lexapro	
Fluoxetine	Prozac, Sarafem	
Paroxetine	Paxil	
Sertraline	Zoloft	
*Venlafaxine	Effexor	
**Atypical Antidepressants**		
Bupropion	Wellbutrin	Animal studies show no clear evidence of teratogenic effects, but there is evidence of a higher pup mortality rate, and lower birth weights, at >MRHD. There are no adequate and well-controlled studies in pregnant women.
Mirtazapine	Remeron	
Nefazodone	Serzone	
Trazodone	Deseryl, Oleptro	
Vortioxetine	Trintellix	
**BENZODIAZEPINES**		
*General FDA warning: First trimester exposure linked to an increased risk of congenital malformations has been suggested in several studies. Non-teratogenic risks include reports of neonatal flaccidity, respiratory and feeding difficulties, hypothermia, and neonatal withdrawal symptoms during the postnatal period. Use of these drugs is rarely a matter of urgency, so first trimester exposure should almost always be avoided.*		
Alprazolam	Xanax	Animal studies suggest risk for teratogenic effects; malformations (cleft palate), have been observed: temazepam has caused exencephaly and fusion or asymmetry of ribs, and is contraindicated in women who are or may become pregnant. Patients should be instructed to discontinue this drug prior to becoming pregnant.
Clonazepam	Klonopin	
Diazepam	Valium	
Lorazepam	Ativan	
Oxazepam	Serax	
Temazepam	Restoril	
**BETA-BLOCKERS**		
Propranolol	Inderal	In animal studies, use of propranolol at dosages at >MRHD caused embryotoxicity and neonatal toxicity. There are no adequate and well-controlled studies in pregnant women. Intrauterine growth retardation, small placentas, and congenital abnormalities have been reported in neonates whose mothers received propranolol during pregnancy. Atenolol use, especially in the 2nd trimester, is associated with infants small for gestational age. Studies with first trimester use are limited.
Atenolol	Tenormin	

**Table 4:** Drugs approved by the FDA to treat bipolar and psychiatric disorders according to drug class and trade name used in the U.S., along with a summary of label information.

ANTIPSYCHOTICS—Typical* and Atypical/Second Generation		
***General FDA warning: Third trimester exposure increases risk for neonatal extrapyramidal and/or withdrawal symptoms (EPS), including reports of agitation, hyper-/hypotonia, tremor, somnolence, respiratory distress and feeding problems. Severity varies from self-limited symptoms to intensive care unit support and prolonged hospitalization.***		
*Haloperidol	Haldol	No teratogenic effects or fetal toxicity have been observed in animal studies involving exposure to clozapine or lurasidone. At doses >MRHD: ziprasidone caused cardiovascular malformations, quetiapine—lower fetal weights and delays in skeletal ossification; aripripazole–increased fetal/pup death, lower birth weight, and skeletal abnormalities. At >MRHD asenapine—lower pup weights and pup mortality, ziprasidone—developmental delays and neurobehavioral impairment. There are no adequate and well-controlled studies in pregnant women. Olanzapine has been associated with adverse pregnancy outcomes, including neonatal death due to cardiovascular defect, and abortion (3 therapeutic, 1 spontaneous).
Aripripazole	Abilify	
Asenapine	Saphris	
Clozapine	Clozaril	
Lurasidone	Latuda	
Olanzapine	Zyprexa	
Rispiridone	Risperdal	
Quetiapine	Seroquel	
Ziprasidone	Geodon	
**MOOD STABILIZERS/ ANTIEPILEPTIC DRUGS**		
Lithium	Eskalith, Lithobid	There are no adequate and well-controlled studies in pregnant women. Lithium may cause Ebstein’s anomaly. Carbamazepine is associated with risk to the fetus, including congenital malformations (spinal bifida), and developmental delays. Valproate may produce congenital malformations (e.g., neural tube defects) at a rate higher than other antiepileptic drugs; other complications include neonatal hepatic failure and hypoglycemia; long-term effects include low IQ and a greater risk for autism spectrum disorderin children. Valproate should not be used to treat women with epilepsy who are pregnant or who plan to become pregnant. If a woman becomes pregnant while taking trimethadione, termination of the pregnancy should be considered. Trimethadione and phenytoin may be associated with a neonatal coagulation defect that may cause bleeding during the early neonatal period (prophylactic Vitamin K may be indicated). Prenatal exposure to phenytoin is associated with a greater risk of neuroblastoma. Risk of use of this class of medications appears particularly high in the 1st trimester. However, abrupt discontinuation of antiepileptic drugs in mothers who use them to prevent major seizures should be avoided as this also creates risk.
Phenytoin	Dilantin	
Phenobarbital	Luminal	
Valproate	Depakote	
Trimethadione	Tridione	
Levitiracetam	Keppra	
Carbemazepine	Tegretol	
Lamotrigine	Lamictal	
**SEDATIVES AND HYPNOTICS**		
Eszopiclone	Lunesta	In animal studies, there is no evidence of teratogenic effects. Offspring of rats exposed to doses higher than the MRHD showed some evidence of delayed ossification, and decreased pup weights/survival. Fewer adverse effects have been found in studies using rabbits. There are no adequate and well-controlled studies in pregnant women. Cases of severe neonatal respiratory depression have been reported when Zolpidem was used at the end of pregnancy, especially when taken with other CNS-depressants
Zaleplon	Sonata	
Zolpidem	Ambien	
